# Acute Effects of Hypoxic Gas Admixtures on Pulmonary Blood Flow and Regional Oxygenation in Children Awaiting Norwood Palliation

**DOI:** 10.7759/cureus.5693

**Published:** 2019-09-18

**Authors:** Lisha Thomas, Saul Flores, Joshua Wong, Rohit Loomba

**Affiliations:** 1 Cardiology, Advocate Children's Hospital, Chicago, USA; 2 Cardiology, Texas Children's Hospital, Houston, USA; 3 Pediatric Cardiology, Advocate Children's Hospital, Chicago, USA

**Keywords:** subambient, norwood, single, ventricle, hypoplastic, left, heart, syndrome

## Abstract

Introduction

Oxygen delivery in patients with functionally univentricular hearts awaiting Norwood palliation depends on a balance between systemic blood flow (Qs) and pulmonary blood flow (Qp). Modulations of pulmonary vascular resistance and systemic vascular resistance are utilized to maintain balanced Qp:Qs in a circulation prone to pulmonary overcirculation at the expense of systemic perfusion. This study aimed to characterize changes in Qp:Qs and regional (cerebral and renal) oxygen delivery in patients awaiting Norwood palliation receiving hypoxic gas admixture therapy.

Methods

Patients who received care prior to Norwood palliation were identified from 2014 to 2018. Of these patients, those with cerebral and renal near-infrared spectroscopy were identified (NIRS). Arterial oxygen saturation by pulse oximetry, renal NIRS, and cerebral NIRS prior to hypoxic gas admixture initiation were compared to values six hours, 12 hours, and 24 hours after initiation.

Results

A total of 28 patients were eligible for inclusion in the study. Arterial saturation by pulse oximetry was 91% prior to initiation and decreased to 86% 24 hours after initiation (p<0.001). Cerebral NIRS were a mean of 60 prior to initiation compared to 58 at 24 hours (p=0.187). Renal NIRS were a mean of 60 prior to initiation compared to 57 at 24 hours (p=0.120). Calculated Qp:Qs was 9.6 at baseline compared to 2.5 at 24 hours (p=0.006). Arteriovenous difference and lactate did not significantly change with hypoxic gas admixture administration.

Conclusion

Administration of hypoxic gas admixture to patients with functionally univentricular hearts awaiting Norwood palliation decreases the ratio of Qp and Qs but does not improve regional oxygenation delivery.

## Introduction

Patients with functionally univentricular hearts undergo staged palliation which often consists of a Norwood palliation, Glenn palliation, and then a Fontan palliation. Both the period prior to the Norwood and Glenn palliation are characterized by parallel circulation in which both the pulmonary and systemic circulations are receiving blood with the same saturation. There is a delicate balance between the pulmonary blood flow (Qp) and systemic blood flow (Qs) which is affected by the pulmonary and systemic vascular resistances. Changes in Qp:Qs may impair systemic oxygen delivery, and thus it may be necessary to modulate Qp:Qs to optimize systemic oxygen delivery [1-3].

Newborns awaiting Norwood palliation will often have pulmonary overcirculation with a high Qp:Qs. Some institutions electively intubate patients and administer a hypoxic gas admixture to increase pulmonary vascular resistance and decrease pulmonary blood flow in hopes of increasing systemic blood flow and systemic oxygen delivery [4-8].

This study aimed to characterize the effects of hypoxic gas administration on Qp:Qs as well as regional oxygen delivery.

## Materials and methods

Study design and patient inclusion

This was a retrospective cohort study utilizing the clinical experience from a single center. The study protocol was approved by the Institutional Review Board. Patients with functionally univentricular hearts who received care prior to Norwood palliation at Advocate Children’s Hospital were identified. To be included in the study final analysis, the following inclusion criteria had to be met: 1) patients had to be awaiting Norwood palliation; 2) patients must not have had any prior interventions other than a balloon atrial septostomy; 3) patients must have received treatment with a hypoxic gas admixture that was administered through an endotracheal or nasotracheal tube using nitrogen; 4) patients must have had cerebral and renal near-infrared spectroscopy (cNIRS and rNIRS) monitoring from the initiation of hypoxic gas admixture administration; 5) hypoxic gas admixture therapy must have continued from its initiation until the Norwood palliation. Thus, those who underwent interventions such as pulmonary artery banding or stenting of the ductus arteriosus were excluded from this study. Patients who were not monitored with near-infrared spectroscopy were also excluded.

Data collection

Demographic data including patient date of birth and date of Norwood palliation were collected. The date and time of the initiation of hypoxic gas admixture were also collected. Specific cardiac anatomy was not collected as this was not thought to affect the physiologic response to the hypoxic gas admixture. The fraction of inspired oxygen at the initiation of hypoxic gas admixture therapy as well as at its lowest point was recorded for each patient.

The following clinical endpoints of interest were collected: arterial saturation by pulse oximetry, the arterial partial pressure of oxygen as measured by blood gas analysis, cNIRS value, rNIRS value, arterial lactate as measured by blood gas analysis, and systolic blood pressure. All of these values were recorded at the following timepoints: immediately prior to the initiation of hypoxic gas admixture (referred to as baseline), six hours after initiation, 12 hours after initiation, and 24 hours after initiation. Only a small number of patients had sub-ambient for greater than 36 hours so the further follow-up was not recorded and analyzed.

The following values were calculated at each timepoint. First, an estimate of pulmonary blood flow (Qp) to systemic blood flow (Qs) ratio was calculated. This will be referred to as the bedside Qp:Qs for the remainder of the text. The calculation of Qp:Qs is as follows: (arterial saturation as measured by pulse oximetry - rNIRS)/(100 - arterial saturation as measured by pulse oximetry). The assumptions for this calculation were: 1) pulmonary artery saturation was assumed to be equal to the arterial saturation as measured by pulse oximetry; 2) pulmonary venous saturation was assumed to be 100%; 3) pulmonary venous saturation was considered to be constant at all time points; 4) rNIRS was used as a surrogate for the mixed venous saturation. Regarding assumption two, chest X-rays at the time of initiation of hypoxic gas admixture were reviewed and were not found to be concerning for pulmonary disease that could lead to pulmonary venous desaturation. Regarding assumption four, there are various proposed calculations for mixed venous saturation using both the cNIRS and rNIRS value in varying proportions. As there was minimal difference between cNIRS and rNIRS values at all timepoints, it was decided to simply use the rNIRS value. Additionally, bedside rNIRS value is more often used clinically compared to the cNIRS. The absolution correlation between the bedside Qp:Qs and catheter-based Qp:Qs is not the purpose of this manuscript. The bedside Qp:Qs is a value which the provider can trend to provide a general overview of the physiology. It is not meant to be used an absolute and certain measure of Qp:Qs. An arteriovenous difference of oxygen (AVDO2) was also calculated at each timepoint. The AVDO2 was calculated for the study as follows: arterial saturation as measured by pulse oximetry - rNIRS.

Statistical analyses

Clinical values collected and calculated at the various timepoints were compared between each timepoint and the timepoint immediately preceding it using paired T-tests. Additional comparisons were conducted between the baseline and 12-hour timepoint as well as the baseline and 24-hour timepoint.

Continuous variables are represented as mean and standard deviation. All statistics were done utilizing the statistical package for social sciences (SPSS), version 23 (IBM Inc., Armonk, NY). A p-value of less than 0.05 was statistically significant.

Clinical care

At our institution, most neonates awaiting Norwood palliation are cared for in the neonatal intensive care unit with both the neonatology and cardiology teams co-managing these patients. Umbilical venous and arterial access is obtained in all patients if possible. A prostaglandin E1 infusion is started immediately once central access is obtained with initial doses ranging from 0.01 mcg/kg/min to 0.03 mcg/kg/min. Respiratory support is titrated as needed and is most often noninvasive. Inotropes are rarely required preoperatively as are diuretics. In the instance that inotropes are needed, dopamine, milrinone, and epinephrine are the most frequently used. If diuretics are required furosemide and chlorothiazide are the most frequently used. Children are allowed to orally feed as much as they can tolerate with close monitoring of systemic perfusion and respiratory status. Nutrition is otherwise supplemented with total parenteral nutrition.

Routine monitoring consists of transduction of the arterial line for blood pressure monitoring and strict monitoring of intake and output. Central venous pressure is not routinely transduced and the use of cNIRS and rNIRS is at the discretion of the clinical team.

Administration of a hypoxic gas admixture has been utilized at the institution nearly routinely in such patients for several years. All patients are electively intubated 24-48 hours prior to scheduled Norwood procedure for hypoxic conditioning. For patients deemed to have high Qp:Qs and clinical signs of systemic hypoperfusion, intubation for hypoxic gas admixture administration is done at the discretion of the clinical team.

## Results

Cohort information

A total of 28 patients met the inclusion criteria described above and were included in the final analyses. The average age at the initiation of hypoxic gas admixture was 3.5 days with the average age at Norwood palliation being 6.1 days. This resulted in an average duration of hypoxic gas admixture administration of 2.6 days. The average fraction of inspired oxygen at the time of initiation was 17% with an average minimum of 15% during hypoxic gas admixture administration (Table 1).

**Table 1:** Baseline cohort characteristics

Characteristics	Mean ± standard deviation
Age at initiation of hypoxic gas admixture (days)	3.5 ± 0.8
Age at Norwood palliation (days)	5.9 ± 1.2
Duration of hypoxic gas admixture (days)	2.4 ± 1.1
Fraction of inspired oxygen at initiation of hypoxic gas admixture	17.3 ± 1.6
Minimum fraction of inspired oxygen	15.7 ± 1.0

Clinical information with hypoxic gas admixture administration

Average baseline pulse oximetry was 91.7%. At six hours this was 90.4%, at 12 hours - 89.8%, and at 24 hours - 86.0%. There was a statistically significant difference between the values at 12 hours and 24 hours as well as prior to initiation and at 24 hours (Table 2).

**Table 2:** Clinical characteristics for those who did and did not receive hypoxic gas admixture (mean ± standard deviation) *Significant difference from timepoint immediately preceding it †Significant difference from baseline (not used if the timepoint immediately preceding it is baseline) Qp - pulmonary blood flow; Qs - systemic blood flow

Characteristics	Prior to initiation	Six hours after initiation	12-hours after initiation	24-hours after initiation
Arterial saturation by pulse oximetry	91.7 ± 6.6	90.4 ± 5.1	89.8 ± 5.2	86.0 ± 6.5*†
Arterial partial pressure of oxygen	45.3 ± 8.3	40.9 ± 5.5*	40.8 ± 7.1†	36.3 ± 5.1*†
Cerebral near-infrared spectroscopy	60.1 ± 12.8	58.5 ± 10.1	61.8 ± 9.4	58.8 ± 9.9
Renal near-infrared spectroscopy	60.5 ± 10.5	59.3 ± 8.9	58.9 ± 13.6	57.9 ± 9.8
Arteriovenous difference	30.8 ± 11.9	31.1 ± 9.7	31.0 ± 13.9	29.6 ± 10.3
Bedside Qp:Qs	9.6 ± 13.0	7.2 ± 9.6	5.1 ± 5.9	3.3 ± 3.6†
Lactate	2.2 ± 1.6	1.6 ± 0.8	1.5 ± 0.6	1.9 ± 1.6
Systolic blood pressure	65.5 ± 9.7	63.0 ± 9.6	65.1 ± 9.7	67.6 ± 8.7*

Average baseline arterial partial pressure of oxygen was 45.3 mmHg. At six hours this was 40.9 mmHg, at 12 hours this was 40.8 mmHg, and at 24 hours was 36.3 mmHg. There was a statistically significant difference between the values at 12 and 24 hours, prior to initiation and 6 hours, prior to initiation and 12 hours, and prior to initiation and 24 hours (Table 2).

Average baseline cNIRS was 60.1. At six hours this was 58.5, at 12 hours this was 61.8, and at 24 hours this was 58.8. There was no statistical significance in the difference between the values at any timepoints (Table 2).

Average baseline rNIRS was 60.5. At six hours this was 58.5, at 12 hours this was 61.8, and at 24 hours this was 58.8. There was no statistical significance in the difference between the values at any timepoints (Table 2).

Average baseline AVDO2 difference was 30.8. At six hours this was 31.1, at 12 hours this was 31.0, and at 24 hours this was 29.6, There was no statistical significance in the difference between the values at any timepoints (Table 2).

Average baseline bedside Qp:Qs was 9.6. At six hours this was 7.2, at 12 hours this was 5.1, and at 24 hours this was 3.3. There was a statistically significant difference between the values prior to initiation and at 24 hours (Table 2).

Average baseline lactate was 2.2 mg/dl. At six hours this was 1.6 mg/dl, at 12 hours this was 1.5 mg/dl, and at 24 hours this was 1.9 mg/dl. There was no statistically significant difference between the values at any time points (Table 2).

Average baseline systolic blood pressure was 65.5 mmHg. At six hours this was 63.0, at 12 hours this was 65.1, and at 24 hours this was 67.6. The only statistical significance found was noted between the value prior to initiation and the value at 24 hours (Table 2).

## Discussion

This study finds that administration of a hypoxic gas admixture to those with functionally univentricular hearts awaiting Norwood palliation is associated with decreased arterial saturation and Qp:Qs as demonstrated by pulse oximetry and arterial partial pressure of oxygen but is not associated with increased oxygen delivery as witnessed by cNIRS, rNIRS, arteriovenous difference, and lactate values.

Balance of the pulmonary and systemic blood flow in the setting of parallel circulation in those awaiting Norwood palliation is of great importance. Without a change in cardiac output, any change in pulmonary or systemic blood flow will lead to an obligate reciprocal change to the other circulation’s blood flow. Thus, an increase in pulmonary blood flow will be at the expense of systemic blood flow and can be problematic if the systemic blood flow becomes inadequate with respect to systemic oxygen delivery [2-3].

As pulmonary vascular resistance naturally decreases after birth, those with parallel circulation have an increase in Qp:Qs. Maintaining a balance of the two circulations can be done by modulation of pulmonary vascular resistance and systemic vascular resistance. Modulation of pulmonary vascular resistance, specifically increasing it, can be difficult. Inhaled hypoxic gas admixture has been used to increase pulmonary vascular resistance. Anecdotally, fewer institutions are now focusing primarily on increasing pulmonary vascular resistance although there are still some who intubate patients to administer a hypoxic gas admixture [4-5]. Our institution is one such institution.

As noted by the presented data, Qp:Qs is in fact significantly decreased after 24 hours of hypoxic gas administration. Systemic oxygen delivery, however, seems to be unchanged. Both cNIRS and rNIRS remain unchanged as did the arteriovenous difference. Lactate levels also remain unchanged which is consistent with the lack of significant change in the NIRS. Systolic blood pressure difference before and after, although statistically significantly higher at 24 hours at a difference of 2 mmHg, was not clinically significant.

Without an improvement in oxygen delivery, the decrease in Qp:Qs may not provide any true clinical advantage. This illustrates the concept that if the total of pulmonary and systemic blood flow is high then systemic blood flow may be adequate even in the setting of a high Qp:Qs. This study demonstrates that oxygen delivery can remain adequate at a Qp:Qs of 9.6:1 and 3.3:1. If systemic oxygen delivery can be maintained at a high range of Qp:Qs, then the clinical utility of altering Qp:Qs must be questioned. If the physiology is being well tolerated then elective intubation of a child awaiting Norwood palliation simply for the purpose of hypoxic gas administration may not be necessary.

In the situation where a child with parallel circulation demonstrates pulmonary overcirculation and poor systemic oxygen delivery, perhaps would it be more beneficial to attempt to lower systemic vascular resistance first [9-10]. Certainly, this would be a noninvasive means of balancing the two circulations and would not preclude later use of hypoxic gas admixture if needed. Maintaining adequate hematocrit in these patients can also help maintain systemic oxygen delivery. Maintaining a target hematocrit with the use of blood transfusions also has the transient effect of increasing pulmonary vascular resistance in relation to systemic vascular resistance [11].

Ramamoorthy and colleagues looked at the effects of inspired hypoxic and hypercapnic gas admixtures on cerebral oxygen saturation in neonates with univentricular heart defects. Even at a fraction of inspired oxygen of 17% there was no significant change in cerebral oxygenation or mean arterial pressure [4]. Tabbutt and colleagues also found similarly that a fraction of inspired oxygen of 17% decreased the arterial partial pressure of oxygen but did not positively impact systemic hemodynamics or oxygenation [5].

This study demonstrates that hypoxic gas admixtures do not negatively impact oxygen delivery while other studies have demonstrated that hyperoxic gas admixtures also do not negatively impact oxygen delivery by increasing Qp:Qs [12]. Thus, the notion of the direct effect of Qp:Qs, in isolation, on systemic oxygen delivery needs to be discarded. These findings highlight that clinical care must be based on an aggregate of clinical data, a critical part of this which is venous saturation, for which NIRS monitoring provides a real-time, noninvasive, and cost-effective surrogate.

The findings of this study echo previously published findings but also add the additional components of rNIRS and lactate. While the data is helpful it is not without its limitations. Obviously, this is a single center study and there could be elements not accounted for that could limit the data’s generalizability. This seems to be less of an issue as components of the findings have been demonstrated before. Another limitations comes in the form of the bedside Qp:Qs calculations. These should not be taken to be accurately measured Qp:Qs but rather a bedside estimation that can often be clinically helpful to trend. To account for this, we have also included absolute data for arterial saturation by pulse oximetry and arterial partial pressure of oxygen.

An additional consideration with the study is that many centers have now discontinued utilizing hypoxic gas admixtures. There are centers in developing countries and even some in developed countries that still continue to use this therapy. Thus, it does become necessary to evaluate contemporary data regarding the intervention. While specific centers may believe the use of hypoxic gas mixtures is now out of vogue it is still used internationally.

## Conclusions

In this single-center retrospective study characterizes the effects of administering a hypoxic gas admixture to functionally univentricular children with parallel circulation awaiting a Norwood procedure. Administration of hypoxic gas admixture to children with functionally univentricular hearts awaiting Norwood palliation decreases the ratio of pulmonary to systemic blood flow but does not improve systemic oxygen delivery as evidenced by near-infrared spectroscopy data and lactate. The lack of improvement in systemic oxygen delivery brings into the question of the utility in decreasing the ratio of pulmonary to systemic blood flow using a hypoxic gas admixture as the purpose of this decrease of the ratio is to ultimately help improve systemic oxygen delivery.

## References

[1] Roeleveld PP, Axelrod DM, Klugman D, Jones MB, Chanani NK, Rossano JW, Costello JM (2018). Hypoplastic left heart syndrome: from fetus to fontan. Cardiol Young.

[2] Austin EH, Santamore WP, Barnea O (1994). Balancing the circulation in hypoplastic left heart syndrome. J Cardiovasc Surg.

[3] Barnea O, Austin EH, Richman B, Santamore WP (1994). Balancing the circulation: theoretic optimization of pulmonary/systemic flow ratio in hypoplastic left heart syndrome. J Am Coll Cardiol.

[4] Ramamoorthy C, Tabbutt S, Kurth CD (2002). Effects of inspired hypoxic and hypercapnic gas mixtures on cerebral oxygen saturation in neonates with univentricular heart defects. Anesthesiology.

[5] Tabbutt S, Ramamoorthy C, Montenegro LM (2001). Impact of inspired gas mixtures on preoperative infants with hypoplastic left heart syndrome during controlled ventilation. Circulation.

[6] Shime N, Hashimoto S, Hiramatsu N, Oka T, Kageyama K, Tanaka Y (2000). Hypoxic gas therapy using nitrogen in the preoperative management of neonates with hypoplastic left heart syndrome. Pediatr Crit Care Med.

[7] Bailey LL, Gundry SR (1990). Hypoplastic left heart syndrome. Pediatr Clin North Am.

[8] Feinstein JA, Benson DW, Dubin AM (2012). Hypoplastic left heart syndrome: current considerations and expectations. J Am Coll Cardiol.

[9] Hoffman GM, Tweddell JS, Ghanayem NS, Mussatto KA, Stuth EA, Jaquis RD, Berger S (2004). Alteration of the critical arteriovenous oxygen saturation relationship by sustained afterload reduction after the Norwood procedure. J Thorac Cardiovasc Surg.

[10] Nakazawa M, Takao A, Shimizu T, Chon Y (1983). Afterload reduction treatment for large ventricular septal defects. Dependence of haemodynamic effects of hydralazine on pretreatment systemic blood flow. Br Heart J.

[11] Lister G, Hellenbrand WE, Kleinman CS, Talner NS (1982). Physiologic effects of increasing hemoglobin concentration in left-to-right shunting in infants with ventricular septal defects. N Engl J Med.

[12] Bradley SM, Atz AM, Simsic JM (2004). Redefining the impact of oxygen and hyperventilation after the Norwood procedure. J Thorac Cardiovasc Surg.

